# Effectiveness of 6-Week Nordic Walking Training on Functional Performance, Gait Quality, and Quality of Life in Parkinson’s Disease

**DOI:** 10.3390/medicina56070356

**Published:** 2020-07-17

**Authors:** Justyna Szefler-Derela, Michal Arkuszewski, Andrzej Knapik, Dagmara Wasiuk-Zowada, Agnieszka Gorzkowska, Ewa Krzystanek

**Affiliations:** 1 Department of Physiotherapy, School of Health Sciences in Katowice, Medical University of Silesia, 40-055 Katowice, Poland; jszefler@sum.edu.pl (J.S.-D.); dwasiuk@sum.edu.pl (D.W.-Z.); 2 Institute for Innovative Medicine, 15-082 Białystok, Poland; michal.arkuszewski@gmail.com; 3 Department of Adapted Physical Activity and Sport, School of Health Sciences in Katowice, Medical University of Silesia, 40-055 Katowice, Poland; aknapik@tlen.pl; 4 Department of Neurology, Faculty of Medical Sciences in Katowice, Medical University of Silesia, 40-055 Katowice, Poland; a_gorzkowska@wp.pl

**Keywords:** Parkinson’s disease, disability, gait, Nordic Walking, rehabilitation exercise, quality of life

## Abstract

*Background and objectives:* Motor rehabilitation improves physical mobility and quality of life in Parkinson’s disease (PD). As specialized rehabilitation is expensive and resource-consuming, there is a need for simpler, cost-effective methods. The purpose of the study was to determine whether Nordic Walking (NW) training may support the management of motor disability in PD. *Materials and Methods:* Forty patients (median age 64.0 years, range 50–75 years) with idiopathic PD, Hoehn and Yahr stages II–III, were randomly assigned to NW or standard rehabilitation (SR) programs, comprising twelve rehabilitation sessions conducted bi-weekly throughout the 6-week study period. *Results:* Median Unified Parkinson’s Disease Rating Scale part III scores were significantly reduced with NW, by 8.5, and with SR, by 6.0 points (both *p* < 0.001), with significantly greater improvement with NW than with SR (*p* = 0.047). Gait quality and balance control, measured using the Dynamic Gait Index, improved with NW by a median of 8.0 and with SR by 5.5 points (both *p* < 0.001), with slightly greater improvement with NW, compared to the SR group (*p* = 0.064). Quality of life, assessed using the Parkinson’s Disease Questionnaire (PDQ-39), improved with NW by a median of 15 and with SR by 12 points, *p* = 0.001 and *p* = 0.008, respectively. *Conclusions:* The 6-week Nordic Walking program improves functional performance, quality of gait, and quality of life in patients with PD and has comparable effectiveness to standard rehabilitation.

## 1. Introduction

Parkinson’s disease (PD) is one of the most common neurodegenerative disorders, affecting 1% of the population above the age of 60 years and 4% of the population above the age of 80 years [1]. Increasing dopaminergic deficits in PD result in bradykinesia, resting tremor, rigidity, and postural instability, which inevitably lead to general motor impairment. Dopaminergic treatment reduces motor symptoms of PD; however, it has to be supported by constant active motor rehabilitation to maintain patients’ physical fitness necessary for their independence [2,3,4].

Regular motor rehabilitation in PD was shown to reduce disease symptoms and improve physical fitness and quality of life [5,6]. Various types of exercises, diverse methods, and different activities are utilized in patients with PD [7]. Most of these rehabilitation programs, however, require individual physiotherapist supervision and, as such, can only be delivered in specialist physiotherapy clinics, which may be expensive and resource-consuming [2,8,9,10]. Therefore, there is a need for simpler, inexpensive alternatives, especially in countries with limited access to rehabilitation services.

Several different patterned exercise methods were proposed for motor rehabilitation in patients with PD [11,12,13,14,15], but a general conclusion on their efficacy is always difficult because of different approaches or different local standards. Nordic Walking (NW) is a sport-walking activity that combines active use of the trunk and upper limbs with traditional walking. It is easy to learn, straightforward to execute, economical, and low-risk as an intervention for PD [16]. The use of poles and upper body rhythmic movements act as external cues to restore movement control, triggering intact circuits and bypassing the defective basal ganglia [3,17,18,19]. Patients may work on improving their stride length, gait variability, maximal walking speed, or balance, all of these being PD-specific limitations [20]. Furthermore, NW can be managed in almost every location and in groups, thus fostering an active lifestyle and social engagement in patients with PD [21,22,23,24]. Therefore, we aimed to determine the clinical effectiveness of a 6-week Nordic Walking rehabilitation program in patients with PD, in comparison to standard rehabilitation (SR).

## 2. Materials and Methods

Forty consecutive patients with idiopathic PD (IPD), diagnosed according to the UK Parkinson’s Disease Society Brain Bank (PDSBB) clinical diagnostic criteria [25] and Hoehn and Yahr (H–Y) disease stages II–III [26], selected from an outpatients’ database in our center, were recruited to this prospective study. PDSBB clinical criteria for IPD include typical clinical presentation, good response to levodopa, and a full differential diagnosis, also involving neuroimaging techniques (CT or MRI). Patients with dementia (Mini-Mental State Examination, MMSE < 24) [27], severe motor fluctuations, freezing, orthostatic hypotension, disabling dyskinesia, severe depression, or other medical conditions significantly affecting mobility or ability to exercise were excluded. Every patient had an optimized treatment with oral drugs, without changes within at least four weeks prior to enrolment, and there was no previous rehabilitation.

Recruited participants were randomly assigned to either of the two groups, NW or SR. The randomization scheme controlled for participant gender to maintain equal within-group and between-group sex distribution.

Each patient from the NW and SR groups participated in 12 rehabilitation sessions conducted bi-weekly over a period of six weeks. All participants were advised to keep up with their usual daily activities at an intensity identical to the one from before the enrolment. A 70% session completion rate was assumed to be sufficient for data inclusion in analyses.

Each NW group session, supervised by a physiotherapist with qualification in NW, was delivered outdoors (in a park). Sessions lasted 90 min and consisted of a warm-up (5–10 min) and specialized NW training aimed at improving walking intensity and distance (60 min), followed by cooling down and stretching (5–10 min). Each individual SR session, supervised by a physiotherapist, was delivered indoors in the rehabilitation facility. SR sessions lasted 45 min and consisted of individually tailored, standard, general-purpose exercises aimed at improving fine and gross motor skills (stretching, high-amplitude movements), as well as active workouts for muscle strength, flexibility, balance, gait, and transfers [28,29,30,31].

The primary endpoint was the between-group difference in the change of the Unified Parkinson’s Disease Rating Scale (UPDRS) motor (part III) scores [32,33], from baseline to the end of the 6-week intervention. UPDRS is designed to longitudinally monitor Parkinson’s-disease-related disability and impairment. Its part III evaluates motor disability and includes ratings for tremor, slowness (bradykinesia), stiffness (rigidity), and balance. It consists of 27 items, where each response is numerically scored 0–4, with a higher score indicating greater symptom severity. A maximum total score of 108 reflects the highest disability level. We considered a 5-point change in the UPDRS motor (part III) score to be the minimal clinically important change (MCIC), as it was shown to be the most appropriate cut-off score for H–Y stages I to III [34]. The between-group differences in the UPDRS motor score of 2.5 (minimum), 5.2 (moderate) and 10.8 points (large) were considered to be clinically important differences (CID) [35].

The secondary outcome measures included changes in functional performance and quality of life parameters. The Dynamic Gait Index (DGI) [36] and timed up-and-go test (TUG) [37,38] are measures of functional mobility, gait quality, and balance control, which may help to assess the risk of falls in older adults [39]. The DGI, with its 8 task-oriented items, measures gait quality, focusing on an individual’s ability to modify gait in response to task demands—e.g., changing gait speed, pivot turning, or climbing stairs, which are considered practical objectives in patients with PD. The TUG, on the other hand, is a measure of functional mobility. In patients with PD, changes of 2.9 points (13.3%) in DGI and 3.5 s (29.8%) in TUG were considered to be minimal detectable changes (MDC) [40]. We used the DGI cut-off score of 22 points to identify patients with a higher risk of falls, as it enables a fairly accurate discrimination between fallers and “safe ambulators” in patients with PD (AUC = 0.84, sensitivity = 0.89, and specificity = 0.48) [39].

The Parkinson’s Disease Questionnaire (PDQ-39) [41] was used to measure changes in the general health status and quality of life of our participants. It is the most commonly used self-reported, disease-specific health status measure, which ascertains patient difficulties across 8 dimensions of daily living: mobility, activities of daily living (ADL), emotional well-being, stigma of the disease, social support, cognition, communication, and bodily discomfort [10,20,22,42,43,44]. The measure is very subjective but thoroughly reflects the well-being of patients with PD. Changes of −4.72 and +4.22 points in PDQ-39 scores over the study period were considered the minimal clinically important limits of improvement and worsening, respectively (MCIC) [45].

Physiotherapists delivering rehabilitation sessions were blinded to all clinical data. Neurological assessments were performed in the neurological outpatient clinic by two neurologists certified in administering the UPDRS, each with over 10 years of experience in PD management, who were blinded to the participant rehabilitation group assignment. Independent physiotherapists, also blinded to the participant rehabilitation group assignment, performed the DGI and TUG and supervised self-reported PDQ 39 in the physiotherapy clinic on the same day as the neurological assessments. All tests were carried out during the medication ‘‘ON’’ period, and doses of antiparkinsonian medication were stable during the whole study period in every participant.

Statistical analysis was performed with use of SYSTAT 12 software (SPSS science, Chicago, IL, USA). Data were presented as a median with minimum and maximum values or as proportions. Having checked the normality of distribution using the Shapiro–Wilk test, the Mann–Whitney U test was used to analyze the between-group differences. A chi-square or Fisher’s exact test was used to analyze categorical data between studied groups. Changes from baseline within each treatment group were analyzed using the Wilcoxon or McNemar test. A *p* value below 0.05 was considered significant.

The study was approved by the Ethics Committee of the Medical University of Silesia (KNW/0022/KB1/111/I/10), and it conforms to the tenets of the Declaration of Helsinki of 1995. All patients gave written informed consent to participate in the study. The study was performed in the years 2013–2014.

## 3. Results

Forty patients with PD, median age 64.0 (50–75) years (50.0% women), were enrolled in the study and randomly assigned to two equal groups (20 patients each) of different rehabilitation strategy, NW or SR. Each patient completed the entire rehabilitation program. Proportions of men and women were equal within and between groups and there were no age differences between groups. The median age of patients at the onset of the disease was 58.0 (35–72) years and median duration of PD treatment was 6.0 (2–18) years, with no between-group differences. The distribution of cases in H–Y stages II and III was balanced between groups. There were no statistically significant differences in baseline characteristics between groups. The baseline demographic and clinical data are presented in the Table 1.

### 3.1. Baseline Clinical Presentation

The median baseline motor UPDRS part III scores were not statistically significantly different between the NW and SR groups (*p* = 0.516), although, numerically, median UPDRS part III score was three points lower in the NW group than in the SR (24.0 (9–42) vs. 21.0 (9–52), respectively), which was slightly greater than the minimal CID of 2.5 points. The median difference in baseline DGI scores between the NW and SR groups (10.0 (4–23) vs. 14.0 (6–23), respectively) was not statistically significant (*p* = 0.283). The overall percentage of patients at high risk of fall (DGI score < 22) was high (87.5%) at baseline, and there were no significant differences between the NW and SR groups (90.0% and 85.0% of patients at high risk of fall, respectively, *p* = 0.633).

The median baseline TUG results were within normal limits in the NW and SR groups (7.09 s (5.25–10.15 s) vs. 7.49 s (5.09–33.82 s), respectively) and without significant differences between groups (*p* = 0.402). Individually, only one patient in the SR group had a TUG result above 30 s and three patients (two in the SR and one in the NW group) had TUG results slightly above 10 s. Additionally, median baseline PDQ-39 scores were not statistically different between the NW and SR groups (59.5 (8–91) vs. 61.0 (16–103), respectively, *p* = 0.655).

### 3.2. Results after 6 Weeks of Training

**Figure 1:**
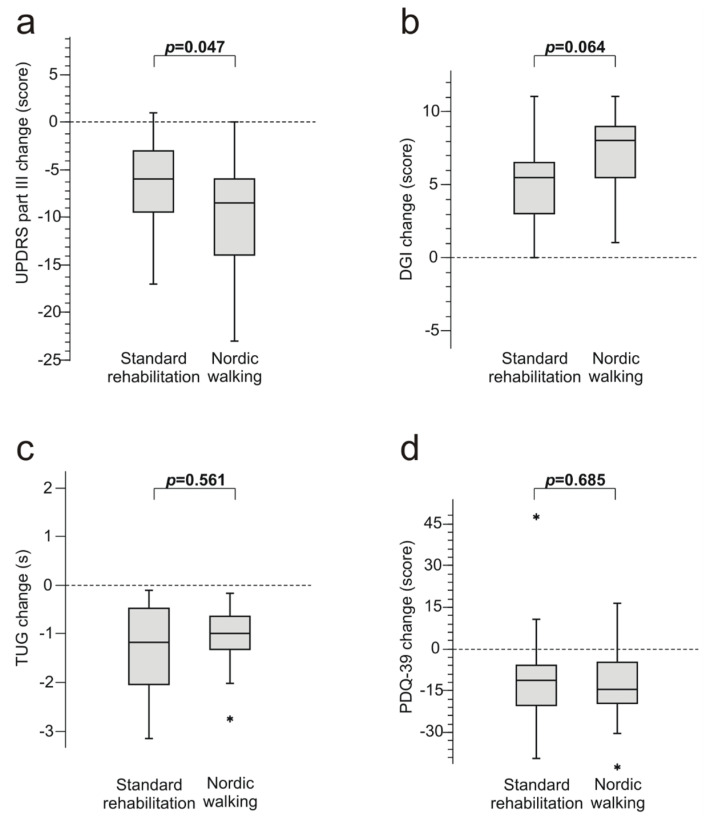
Box and whisker plots of median change in (**a**) Unified Parkinson’s Disease Rating Scale (UPDRS) part III scores, (**b**) Dynamic Gait Index (DGI) scores, (**c**) timed up-and-go test (TUG) results and (**d**) 39-item Parkinson’s Disease Questionnaire (PDQ-39) scores in patients with Parkinson’s disease after 6 weeks of Nordic Walking program or standard rehabilitation (n = 20 in each group). Central line of the box represents the median, top and bottom margins of the box represent the 1^st^ and 3^rd^ quartiles, and the ends of the whiskers represent the minimum and maximum values; significant outliers are presented with asterisks. Pairwise comparisons performed with Mann–Whitney U test.

The median values of UPDRS, DGI, TUG, and PDQ-39 significantly improved in both rehabilitation groups (all differences between baseline and the end of study, *p* < 0.001, except for the change in the SR group in PDQ-39, *p* = 0.008) (Figure 1a). The median improvement in UPDRS part III score exceeded the 5-point MCIC in both rehabilitation groups; therefore, functional improvement in both groups was considered clinically significant. After 6 weeks of the NW program, functional impairment reduction measured by UPDRS part III scores was significantly greater (*p* = 0.047) than after SR (reduction of 8.5 vs. 6.0 points, respectively).

The improvement of gait quality and balance control assessed using the DGI scores was greater than the MDC of 2.9 points in both rehabilitation groups. After 6 weeks of participation in the study, reduction of gait impairment measured with DGI scores was greater in the NW than in the SR group (8.0 vs. 5.5 points, respectively), but the difference was not statistically significant (*p* = 0.064) (Figure 1b). The proportion of patients with a high risk for falls (DGI scores below 22) decreased from 90% to 65% and from 85% to 65% in the NW and SR groups, respectively, and there was no difference between groups in the reduction rate (*p* = 0.705).

The median TUG test results improved slightly in the NW and the SR group (−0.96 s (−2.75 to −0.18) vs. –1.18 s (−3.16 to −0.10), respectively, *p* = 0.561) (Figure 1c). Observed median changes were not considered clinically relevant in either of the two groups, as they did not exceed the MDC of 3.5 s (or 29.8%). Substantial improvements could not be expected, as baseline values were within normal limits (<10 s) in the majority of patients.

Functional improvement in both rehabilitation groups was associated with substantial changes in the quality of life scores measured with the PDQ-39, but there was no difference in the magnitude of change between the NW and SR groups (−15.0 points (−43 to 16) vs. –12.0 points (−40 to 47), respectively, *p* = 0.685) (Figure 1d). The improvements in PDQ-39 scores in the NW and SR groups exceeded the MCIC of 4.72 and therefore were considered clinically relevant. There was no case of injury or fall sustained during the NW training or SR activities.

## 4. Discussion

This study showed that short active motor rehabilitation programs provide substantial functional benefit for patients with PD in improving their quality of life. This supports previously observed positive effects of different rehabilitation methods in PD [46,47]. It was already shown that exercise enhances the efficacy of dopamine substituting compounds, probably as a result of an improved endogenous dopamine release [48]. However, although evidence-based review of treatments for the motor symptoms of PD categorized non-pharmacologic and physiotherapy techniques as “clinically useful”, patterned exercise methods, such as NW, were categorized as only “possibly useful” because of insufficient evidence for their effectiveness alone [7,16]. This study adds to the general understanding of the role of physical exercise in PD because relatively short rehabilitation intervention resulted in a substantial functional improvement without any changes to medical treatment. We have also shown that, being simple and easily accessible, Nordic Walking training may be complementary to standard rehabilitation, as it offers comparable functional results.

Both rehabilitation programs showed comparable efficacy for improvement of functional performance, with the change in UPDRS part III scores exceeding the MCIC of 5 points. The UPDRS scale is widely used in randomized clinical trials evaluating medical treatments for PD [32,33]. Such trials are most often designed to detect treatment effects with significantly longer follow-up, and the MCIC in UPDRS part III was determined in patients receiving treatment for PD for at least 6 months [34]. Our results achieved in UPDRS motor scores are comparable to those reported by others, where mean improvement after the NW rehabilitation program was 6.4 points in one study [20] and 7.5 points in the other [22]. Substantial improvement achieved in both groups after only 6 weeks in our study, and without any additional changes to medical treatment, confirms the high value of motor rehabilitation as an essential part of standard of care in PD.

A significant functional improvement after 6 weeks of rehabilitation was also reflected in both secondary measures, DGI and TUG, which are frequently used for the assessment of treatment-related benefits [49,50,51]. Both rehabilitation strategies resulted in a substantial improvement of DGI scores, which was consistent with the change observed in UPDRS. This was reflected in the change in the proportion of patients at high risk of falls (DGI < 22), which decreased substantially in both study groups and without any change in medication. It confirms previous findings that exercise training can improve balance and gait ability in individuals with PD and decrease their fall rates over both, short- and long-term periods [52].

The TUG was the only stopwatch-based test used in our study. Although a significant improvement was observed in both rehabilitation groups, neither of them hit the MDC threshold of 3.5 s (29.8%); therefore, the change cannot be considered clinically relevant. The TUG and its modifications (TUGFS—timed up-and-go forced speed—and TUGSS—timed up-and-go at self-selected speed) are often used in PD studies, including NW, but the results are not always positive [22,42,43]. Considering that median baseline TUG results in both groups were within normal limits (<10 s) in our study, substantial improvements could not be expected. It seems that stopwatch-based tests may not be the best option for clinical assessment of complex functional limitations in patients with PD, because their results do not closely correlate with other clinical measures. In our study, the DGI better reflected initial gait or mobility limitations and subsequent rehabilitation-related changes than the TUG.

This study showed that functional improvement observed across all measures in both rehabilitation groups (NW and SR) was also reflected in a substantial improvement in quality of life, which is in line with previous observations [43,53]. Positive effects of NW may also be attributable to the social aspect of group training, where interaction with fellow PD patients helps subjects to better accept their condition [54], or to the more indirect beneficial effects of improved mobility on sleep, constipation, mood, or cognition [21]. Quality of life is the principle of well-being perceived by people, and our findings add to the evidence supporting the efficacy of motor rehabilitation in patients with PD.

The value of NW was assessed in several studies in patients with PD, although an analytical review of six randomized controlled trials evaluating the efficacy of NW training in PD [16] (with the biggest NW group being 30 PD patients and study duration being 4, 6, 8, 12, or 24 months) is inconclusive, mainly due to significant methodological heterogeneity between these trials. The studies differed in sample size and the duration of NW training. The study by Monteiro et al. [43] conducted in a similar-sized group, showed that NW improves functional parameters and walking mobility better than the so-called free walk, although their study group was more heterogeneous (H–Y stage I–IV) than ours (H–Y stage II–III).

Several different patterned exercise methods were proposed for motor rehabilitation in patients with PD [11,12,13,14,15]; however, there is no specific guidance on their individual value because of substantial heterogeneity between studies. In our study, the SR sessions were shorter, although 90 min NW sessions included warming up and cooling down, and thus, the time for exercise was comparable. The NW sessions were carried out in the local park, thus not requiring the special scheduling of SR, which had to be delivered in the physiotherapy clinic. The latter required additional commuting time and scheduling logistics. Furthermore, SR, unlike NW, was delivered in individual rather than group sessions, requiring higher involvement of physiotherapy staff at the center. In comparison with SR, NW was easier to access, it was a group activity carried outside, with only one instructor involved at the beginning, and after the initial general practice, no further assistance was needed.

There are some limitations to our study. Our study group was not fully representative for the whole population of patients with PD, as we included only patients with H–Y stages II–III. Patient selection was based on an assumption that the less disabled patients (Hoehn–Yahr stage I) may be less interested in physical activity and the most disabled ones are too physically limited to access NW or SR. We did not intend to verify the clinical diagnosis of IPD with the use of additional neuroimaging techniques, such as dopamine transporter images (PET or SPECT) or MIBG scintigraphy, due to funding limitations. Participants were diagnosed with IPD based on PDSBB clinical diagnostic criteria, the diagnostic accuracy of which is 82% [25]. All inclusion/exclusion criteria were confirmed at the study entry based on all historical data, including neuroimaging documentation (CT or MRI), to exclude possible cerebral abnormalities such as structural lesions in the basal ganglia, multiple small vessel disease, or dilated ventricles, which may affect gait, ADL, or Parkinsonian symptoms. The number of men and women in our study was equal, just as in the study by Reuter et al. [20], which was intentional and forced by our randomization algorithm. We assumed that maintaining gender parity in all groups might help to avoid differences in clinical presentation or different response to physical activity [55]. Unfortunately, this resulted in an imbalanced clinical presentation at baseline as measured with UPDRS and other clinical variables. We used the first published UPDRS [32,33]; therefore, it is difficult to compare our results with other studies using more recent UPDRS versions [56]. The study lacks longitudinal observation, and we cannot assess whether the clinical effect could be maintained after study completion, just as we cannot predict whether the participants will regularly continue their NW activity.

## 5. Conclusions

A 6-week Nordic Walking training program improves functional performance, quality of gait, and quality of life in patients with PD and shows equal effectiveness to standard rehabilitation. Inexpensive and easily accessible, Nordic Walking activity may be complementary in an optimized standard of care for older men and women with PD.

## Figures and Tables

**Table 1:** Baseline demographic and clinical data of patients with Parkinson’s disease assigned to two rehabilitation strategies, Nordic Walking (NW) and standard rehabilitation (SR).

	NW *N* = 20	SR *N* = 20
Women, *n* (%)	10 (50.0)	10 (50.0)
Age, years	62.5 (50–75)	65.5 (54–75)
Age at onset, years	56.5 (35–70)	59.0 (45–72)
Duration of treatment, years	6.0 (3–18)	5.0 (2–14)
Hoehn and Yahr stage, *n* (%)	II, 9 (45.0) III, 11 (55.0)	II, 11 (55.0) III, 9 (45.0)

Note: Unless indicated, values are expressed as a median with minimum and maximum in brackets.
